# Sleep Instabilities Assessed by Cardiopulmonary Coupling Analysis Increase During Childhood and Adolescence

**DOI:** 10.3389/fphys.2018.00468

**Published:** 2018-05-08

**Authors:** Dirk Cysarz, Maijana Linhard, Georg Seifert, Friedrich Edelhäuser

**Affiliations:** ^1^Integrated Curriculum for Anthroposophic Medicine, Institute of Integrative Medicine, University of Witten/Herdecke, Witten, Germany; ^2^Department of Pediatric Oncology/Hematology, Otto-Heubner-Center for Pediatric and Adolescent Medicine, Charité Universitätsmedizin, Berlin, Germany

**Keywords:** cardiopulmonary coupling analysis, childhood and adolescence, sleep electrocardiogram, sleep quality

## Abstract

The electrocardiogram-based cardiopulmonary coupling (CPC) technique may be used to track sleep instabilities. With progressing age, maturational changes during childhood and adolescence affect sleep. The objective was to assess developmental changes in sleep instabilities in a natural setting. ECGs during nighttime sleep on regular school days were recorded from 363 subjects aged 4 to 22 years (204 females). The estimated total sleep time (ETST) decreased from 598 to 445 min during childhood and adolescence. Stable sleep linearly decreased with progressing age (high frequency coupling (HFC): 70–48% ETST). Unstable sleep [low frequency coupling (LFC): 9–19% ETST], sleep fragmentation or disordered breathing (elevated LFC: 4–12% ETST), and wake/REM states [very low frequency coupling (VLFC): 20–32% ETST] linearly increased with age. Hence, with progressing age the sleep of children and adolescents shortens, becomes more unstable and is more often affected by fragmentation or sleep disordered breathing, especially in the age group >13 years. It remains to be clarified whether some of the changes are caused by a social jetlag, i.e., the misalignment of body clock and social time especially in adolescents.

## Introduction

Many physiological functions are associated with age-related changes during childhood and adolescence. Sleep is also considerably altered during this period, e.g., sleep duration declines with progressing age. The recommended sleep duration over 24 h for pre-schoolers is 11–12 h whereas teenagers should sleep 9 h on average (Galland et al., 2012; Hirshkowitz et al., 2015; Paruthi et al., 2016). The decline in sleep duration is accompanied by a considerable delay of sleep time. Preschoolers go to bed early in the evening and wake up early in the morning whereas adolescents go to bed late and wake up later in the morning (on free days) (Roenneberg et al., 2004; Foster and Roenneberg, 2008). This change of sleep preferences is accompanied by a progressive delay of the body clock by up to 2 h during childhood and adolescence. At the same time sleep patterns also change: during non-rapid eye movement (non-REM) sleep, e.g., the amount of slow wave sleep (1–4 Hz delta EEG) decreases whereas the amount of stage 2 sleep increases with progressing age (Scholle et al., 2011; Carskadon and Dement, 2017). At the same time the average amplitude of delta oscillations decreases whereas its mean frequency increases during non-REM sleep (Feinberg and Campbell, 2013). These examples illustrate that sleep is not a static function. Instead, the restorative and regenerative function of sleep depends on age and it obviously adapts to the demands and requirements of the organism at the respective age.

Sleep instabilities during non-REM sleep, as assessed by the occurrence of cyclic alternating patterns (CAP) in the EEG, also show age-related changes during childhood and adolescence (Parrino et al., 2012). The CAP rate, i.e., the percentage ratio of CAP time to non-REM sleep time, is low during infancy and increases with progressing age, shows a peak at puberty and decreases during adolescence. Although the term ‘instability’ may suggest a negative significance, these sleep instabilities are an essential part of the sleep microstructure because they take part in the formation of sleep cycles. Furthermore, the amount of CAP, i.e., the amount of unstable sleep, correlates to cognitive performance: in normal children at the age of 3–12 years, the CAP rate was positively correlated to the capability of non-verbal fluid reasoning (Bruni et al., 2012; Novelli et al., 2013).

The assessment of sleep instabilities in children so far has been based on the analysis of appearance of CAP in polysomnographic data with a relatively low number of subjects, e.g., 10 children (Bruni et al., 2002, 2005). It has been shown that sleep instabilities also have an impact on cardiac autonomic regulation as assessed by spectral analysis of heart rate variability (HRV) (Ferri et al., 2000; Ferini-Strambi et al., 2000). Hence, we focus on the assessment of stable and unstable sleep by means of an electrocardiogram-based technique called cardiopulmonary coupling (CPC) (Thomas et al., 2005). The analysis of CPC quantifies the degree of coherent coupling between HRV and variations of the R-wave amplitude caused by modulations of the respiratory tidal volume. CPC of high frequency oscillations [high frequency coupling, (HFC)] indicates stable sleep, i.e., non-CAP sleep, because vagal modulations induced by respiration (i.e., respiratory sinus arrhythmia) prevail in the cardiac autonomic regulation. Low frequency coupling (LFC) is associated with sleep instabilities, i.e., CAP sleep, because recurring sympathetic arousals lead to recurring short episodes of increased of heart rate in the low frequency range. A special characteristic of LFC, so-called elevated LFC, can be used to detect periods of apnea and hypopnea (Thomas et al., 2007). Furthermore, the extent of HFC is related to the amount of slow wave oscillations in the EEG (Thomas et al., 2014). In a pediatric population, HFC has also been observed to correlate negatively with sleep-disordered breathing (Guo et al., 2011).

The purpose of this study was to assess changes of sleep instabilities during childhood and adolescence assessed by the CPC technique. This technique is applied to ECG data from a larger cohort that we have previously investigated with respect to changes of cardiac autonomic regulation during childhood and adolescence (Cysarz et al., 2011, 2013). The 24-h ECG data were acquired on regular school days and, hence, we assess maturational changes of ECG derived sleep characteristics in a context that highly shaped daily activities.

## Materials and Methods

### Subjects

ECG recordings from a previous study were re-analyzed (Cysarz et al., 2011). Initially, 469 subjects were enrolled in the cross-sectional study. 363 (age range 4–22 years; 204 females, 159 males) subjects had an ECG recording suitable for the nighttime sleep analysis, i.e., a continuous ECG recording free of major artifacts was available for the entire sleep. The subjects were divided into the following four age groups to allow comparisons among the groups: (1) pre-schooler, age <7 years (mean 5.7 ± 0.9 years; *N* = 74, 47 females); (2) primary school children, 7 ≤ age <10 years (mean 8.5 ± 0.8 years; *N* = 99, 54 females); (3) early adolescence, 10 ≤ age < 14 years (mean 11.8 ± 1.1 years; *N* = 89, 48 females); and (4) adolescence, age >14 years (mean 17.8 ± 2.2 years, *N* = 101, 55 females). This age classification is closely related to an age based classification using clinical parameters (Kliegman et al., 2016).

None of the subjects had any history of cardiovascular disease. With respect to sleep disturbances 4 subjects had diseases (e.g., bronchial asthma) that could have affected sleep. Five subjects were taking medication for attention-deficit hyperactivity disorder and four subjects were receiving naturopathic treatment which may have affected sleep. A *post hoc* analysis comparing the results of the entire group and the results of the groups without the above-mentioned subjects showed no differences. Hence, all subjects were included in the analysis.

### Ethics Statement

Written informed consent was obtained from the child’s guardian and, if applicable, also from the child in accordance with the Declaration of Helsinki. The study protocol was approved by the Ethics Committee of Charité – Universitätsmedizin Berlin.

### ECG Recordings

Twenty-four hour-Holter ECGs were recorded under regular school day conditions during the week. The subjects were introduced to aim of the ECG recording and the technical handling of the device. Furthermore, information for parents (or legal guardians) was provided to support the handling. Subsequently, they were equipped with the Holter device at school in the morning and the recording was stopped the next morning. The digital Holter device (Medilog MK3, Schiller-Engineering, Graz, Austria) had a built-in R-peak detection with a precision of <1 ms (internal sampling rate: 4096 Hz). The ECG (at a sampling rate of 256 Hz) as well as the automatically identified times of the R-peaks were transferred to a PC for further analysis. The times of the automatically identified R-peaks were inspected manually using the saved ECG. In case of ventricular extrasystoles, artifacts and premature beats the timings of the related beats were marked accordingly (<0.5% of all R-peaks). Times of normal beats that were not correctly identified by the device’s built-in R-peak detection were corrected manually on the basis of the saved ECG. Hence, the times of these corrected beats had a precision of 4 ms (<1.5% of all R-peaks).

### Cardiopulmonary Coupling Analysis

The nighttime sleep period was part of the 24 h-ECG recordings. Bedtime and wake-up time had to be noted in a diary by the subjects or their legal guardians. In many cases these diary sleeping times were not available. Hence, in order to consistently assess sleep times, the following procedure was used to detect sleep onset and end of sleep. It has been shown that cardiac autonomic regulation clearly changes during the onset of sleep. The average RR-interval, the very low frequency component of HRV and the ratio of the low frequency component to the high frequency component, LF/HF, decrease during the onset of sleep (Trinder et al., 2001; Shinar et al., 2006; Kuo et al., 2008). Using a customized program, two diagrams were displayed one above the other. In one diagram, the series of RR-intervals and its 30 s moving average was plotted versus time (i.e., the occurrences of R-peaks). In a second diagram, the ratio LF/HF was plotted for each successive 30 s interval. A clear increase of RR-intervals (i.e., a drop of heart rate) accompanied by a clear decrease of LF/HF in the evening was defined as the time of onset of sleep. In rare cases only LF/HF showed a clear decrease in the evening whereas the clear increase of the RR-intervals was delayed. In these cases the timing of decrease of LF/HF was evaluated as the onset of sleep. The wake-up time was defined as a clear decrease of RR-intervals in the morning accompanied by a clear increase of LF/HF. This procedure could only estimate the total sleep time and is, hence, termed ‘estimated total sleep time’ (ETST).

The analysis of CPC relies on cross-spectral and coherency estimates between variations of heart rate and its associated changes of tidal volume, i.e., ECG-derived respiration (EDR). The EDR signal consisted of the series of R-peak amplitudes (Moody et al., 1985, 1986). Two properties need to be fulfilled for CPC: (1) both signals need to oscillate at a given frequency. This is quantified by cross-spectral analysis. (2) The signals need to be coupled and synchronized. This property is assessed by the analysis of coherence, i.e., the phases of both signals must be aligned so that the phase relationship is constant. The product of these two properties is the key in the quantification of the degree of CPC (Thomas et al., 2005).

All calculations were carried out as recently described to assure comparability of the results with findings of previous studies (Thomas et al., 2005). First, outliers in the RR-interval series were removed. The RR-interval series and its EDR signal were re-sampled at 2 Hz using cubic spline interpolation. The cross-spectral power and the coherence of these two signals was calculated of a 1024 sample window (approx. 8.5 min) using the Fourier transformation. For each analysis window the sum of the two maximal coherent cross-power peaks in the very low frequency band (0–0.01 Hz), the low frequency band (0.01–0.1 Hz) and the high frequency band (0.1–0.4 Hz) was used to assess physiological couplings. HFC was associated to respiratory sinus arrhythmia and deep sleep. This is termed stable sleep and is linked to non-CAP sleep. On the other hand, the presence of LFC is associated with CAP sequences, i.e., unstable sleep. Furthermore, wake/REM periods are associated to the appearance of very low frequency coupling (VLFC). Using appropriate thresholds, sleep can be categorized into HFC (stable sleep), LFC (unstable sleep), and VLFC (wake/REM state). We note that the detection thresholds varied across studies dealing with CPC (Thomas et al., 2005, 2009; Ibrahim et al., 2010). Here, we used the thresholds that were established in the initial study (Thomas et al., 2005).

Sleep instabilities caused by sleep disordered breathing are associated with an elevated low frequency coupling (e-LFC), a subset of LFC (Thomas et al., 2007). We also assessed the amount of e-LFC of each ECG recording. VLFC, LFC, HFC, and e-LFC were calculated as ratio of the total duration of each state to the ETST. The percentage ratio of the changes of the sleep states VLFC, LFC, and HFC between successive analysis windows to the total number of analysis windows was termed ‘sleep fragmentation.’ A lower percentage ration indicates fewer changes of sleep states, i.e., a less fragmented sleep.

In addition, the CAP-rate and the proportion of non-REM sleep were assessed using VLFC, LFC, and HFC. The CAP-rate, i.e., the percentage ratio of the total CAP time to non-REM sleep time, is assessed as the percentage ratio of the LFC time to the combined LFC and HFC time. The proportion of non-REM sleep time (time spent in sleep stages N1–N3) to the total sleep time is estimated by the proportion of the combined HFC and LFC time, i.e., time spent in stable and unstable non-REM sleep, to the ETST. All calculations were carried out using customized Matlab programs.

### Statistical Analysis

Each parameter was plotted vs. age to describe the dependency on age. The average time course was plotted by means of a moving average (window length: 21 data points). The dependency of the parameters on age was modeled by a regression analysis using a 3rd order polynomial. The significance of this modeling was calculated using the F-statistic.

The results of the four age groups are presented as mean ± SD. The parameters were normally distributed as indicated by the Kolmogorov–Smirnov test (*p* < 0.01). Comparisons between the groups were calculated using the 1-way ANOVA procedure. The *post hoc* comparisons were Bonferroni corrected. Student’s *t*-test was used to compare results of male and female subjects. A value *p* < 0.05 was considered statistically significant.

## Results

**FIGURE 1:**
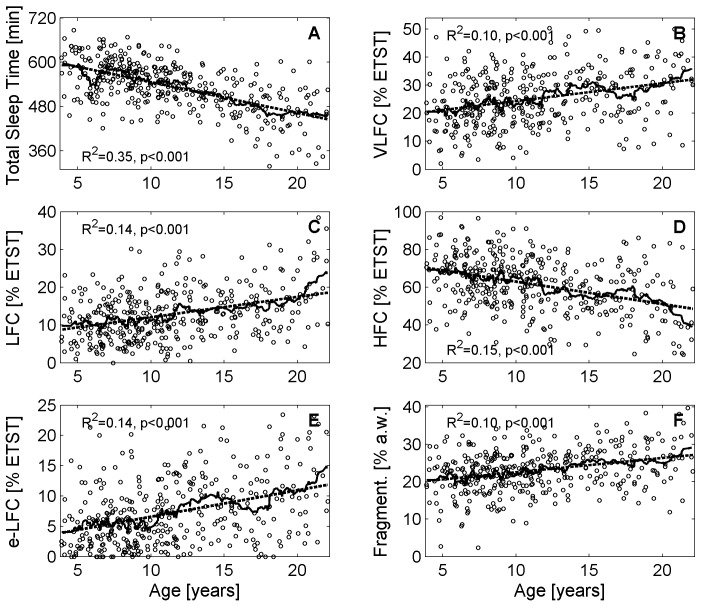
Variations of cardiopulmonary coupling (CPC) parameters during childhood and adolescence. **(A)** Estimated total sleep time (ETST), **(B)** Very low frequency coupling (VLFC) indicating REM/wake states, **(C)** Low frequency coupling (LFC) indicating unstable non-REM sleep, i.e., cyclic alternating pattern (CAP) sleep, **(D)** High frequency coupling (HFC) indicating stable non-REM sleep, i.e., non-CAP sleep, **(E)** Elevated low frequency coupling (e-LFC) indicating disordered breathing, and **(F)** fragmentation of sleep. The solid lines show the moving average (21 consecutive data points) and the dashed line indicates the linear regression model.

**FIGURE 2:**
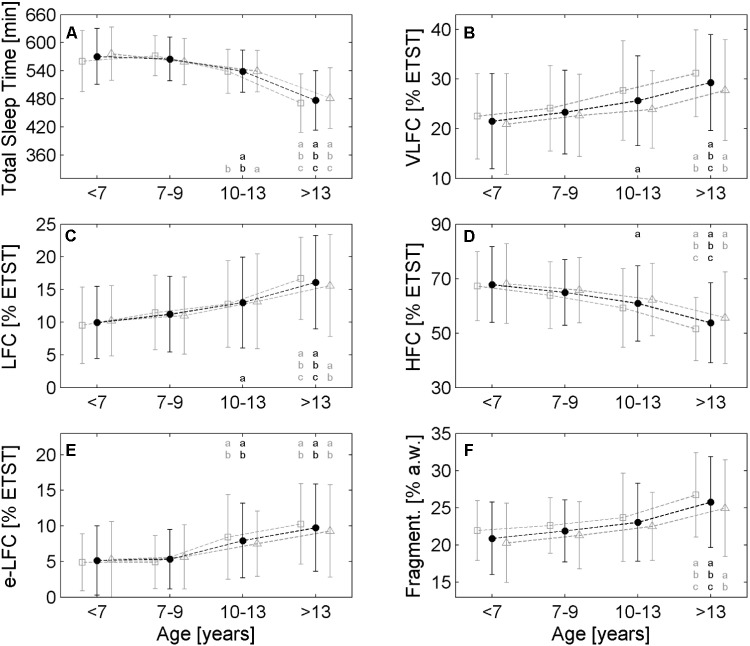
Comparison of different age groups with respect to the cardiopulmonary coupling (CPC) parameters. **(A)** Estimated total sleep time (ETST), **(B)** Very low frequency coupling (VLFC) indicating REM/wake states, **(C)** Low Frequency Coupling (LFC) indicating unstable non-REM sleep, i.e., cyclic alternating pattern (CAP) sleep, **(D)** High Frequency Coupling (HFC) indicating stable non-REM sleep, i.e., non-CAP sleep, **(E)** Elevated low frequency coupling (e-LFC) indicating disordered breathing and **(F)** fragmentation of sleep. Each age group shows three values: the average of the entire group is plotted in the middle (black points), open squares on the left of each black point show the average of male subjects and open triangles show the average of female subjects. The small letters in each diagram refer to significant differences (*p* < 0.05) with respect to the following age group: a: <7 years, b: 7–9 years, c: 10–13 years. Black letters refer to the whole group, gray letters on the left and right of the black letter denote males and females, respectively.

Generally, all investigated parameters showed age related changes and a linear model was sufficient to describe the changes. The average ETST according to the linear model (*R*^2^ = 0.35, *p* < 0.001) was 598 min (10.0 h) for the pre-schoolers and declined to 445 min (7.4 h) at the end of adolescence (see **Figure 1A**). The four age groups reflected this decline (see **Figure 2A**; <7 years: 571 ± 60 min, 7–9 years: 565 ± 47 min, 10–13 years: 539 ± 45 min, >13 years: 477 ± 64 min; *p* < 0.001). The age group >13 years had the shortest ETST compared to the other age groups (*p* < 0.05). Furthermore, also the age group 10–13 years was different to the age group 7–9 years and the age group <7 years (*p* < 0.05).

Very low frequency coupling, i.e., REM/wake periods, was lowest for the pre-schoolers (approx. 20% of the ETST; see **Figure 1B**) and increased to approx. 32% ETST at the end of adolescence (linear model: *R*^2^ = 0.10, *p* < 0.001). The age groups also reflected the increase (see **Figure 2B**; <7 years: 21.5% ± 9.6% ETST, 7–9 years: 23.3% ± 8.4% ETST, 10–13 years: 25.6% ± 9.0% ETST, >13 years: 29.3% ± 9.7% ETST; *p* < 0.001). The age group >13 years had the greatest VLFC compared to all other age groups (*p* < 0.05) and also the coupling in the age group 10–13 years was greater than that of the age group <7 years (*p* < 0.05).

Low frequency coupling, i.e., unstable non-REM sleep, increased with progressing age (see **Figure 1C**). The pre-schoolers showed LFC in approx. 9% ETST whereas LFC was approx. 19% ETST at the end of adolescence (linear model: *R*^2^ = 0.14, *p* < 0.001). The age groups showed an increase of LFC especially for the group >13 years (see **Figure 2C**; <7 years: 10.0% ± 5.5% ETST, 7–9 years: 11.2% ± 5.6% ETST, 10–13 years: 13.0% ± 6.9% ETST, >13 years: 16.1% ± 7.1% ETST; *p* < 0.001). The age group >13 years had the greatest LFC compared to all other age groups (*p* < 0.05) and also the coupling of the age group 10–13 years was greater than that of the age group <7 years (*p* < 0.05).

High frequency coupling, i.e., stable non-REM sleep, decreased with progressing age from approx. 70% ETST for the pre-schoolers to approx. 48% ETST at the end of adolescence (linear model: *R*^2^ = 0.15, *p* < 0.001; see **Figure 1D**). The age groups also showed a decrease (see **Figure 2D**; <7 years: 67.8% ± 13.9% ETST, 7–9 years: 64.9% ± 12.1% ETST, 10–13 years: 60.9% ± 13.8% ETST, >13 years: 53.7% ± 14.7% ETST; *p* < 0.001). The age group >13 years showed a clearly reduced HFC compared to all other age groups (*p* < 0.05). Furthermore, the coupling of the age group 10–13 years was lower compared to that of the age group <7 years (*p* < 0.05).

The estimated proportion of non-REM sleep for the pre-schoolers was approx. 79% of the ETST. At the end of adolescence, the proportion decreased to approx. 67% (linear model: *R*^2^ = 0.10, *p* < 0.001). The age groups reflected this decrease: age group <7 years: 77.8% ± 10.0% ETST, age group 7–9 years: 76.1 ± 8.5% ETST, age group 10–13 years: 73.9% ± 9.3%, age group >13 years: 69.8% ± 10.1%. The age group >13 years had the lowest estimated proportion of non-REM sleep compared to all other age groups (*p* < 0.05).

The estimated CAP-rate increased from 11% of total non-REM time for the preschoolers to 29% of total non-REM time at the end of adolescence (linear model: *R*^2^ = 0.16, *p* < 0.001). Accordingly, the age groups showed an increase with increasing age: <7 years: 13.6% ± 8.7% est. non-REM time, 7–9 years: 15.2% ± 8.5% est. non-REM time, 10–13 years: 18.5% ± 11.1% est. non-REM time, >13 years: 24.2% ± 12.4% est. non-REM time. The age group >13 years showed a clear increase compared to all other age groups (*p* < 0.05) and also the age group 10–13 years was higher compared to the age group <7 years (*p* < 0.05).

Elevated LFC (e-LFC), i.e., fragmented sleep or sleep disordered breathing, increased with progressing age. e-LFC was lowest for the pre-schoolers (approx. 4% ETST) and increased to approx. 12% ETST at the end of adolescence (linear model: *R*^2^ = 0.14, *p* < 0.001; see **Figure 1E**). Accordingly, the age groups also showed an increase of e-LFC (see **Figure 2E**; 5.1% ± 4.9% ETST, 7–9 years: 5.3% ± 4.2% ETST, 10–13 years: 7.9% ± 5.2% ETST, >13 years: 9.7% ± 6.1% ETST; *p* < 0.001). The age group > 13 years and the age group 10–13 years had a higher e-LFC compared to the age groups 7–9 years and <7 years (*p* < 0.05).

The relative number of changes of sleep states between successive analysis windows (a.w.), i.e., sleep fragmentation, also showed an increase with progressing age: 20% changes in relationship to all analysis windows for the pre-schoolers and 27% changes at the end of adolescence (linear model: *R*^2^ = 0.10, *p* < 0.001; see **Figure 1F**). The age groups also reflected the increase of the fragmentation (see **Figure 2F**; 20.9% ± 6.0% a.w., 7–9 years: 21.9% ± 5.1% a.w., 10–13 years: 23.1% ± 4.9% a.w., >13 years: 25.8% ± 5.7% a.w.; *p* < 0.001). The age group >13 years had a higher fragmentation compared to all other age groups (*p* < 0.05).

Gender differences were generally weak. The diagrams in **Figure 2** show slightly higher VLFC, LFC, e-LFC and sleep fragmentation in male subjects compared to female subjects especially for the age group >13 years. On the other hand, HFC and ETST are slightly lower in male subjects compared to female subjects. However, only VLFC showed a clear difference in the age group 10–13 years: male subjects had greater VLFC compared to female subjects (27.7% ± 10.0% ETST vs. 23.9% ± 7.8% ETST, *p* < 0.05). In this age groups the male subjects also tended to a lower proportion of estimated non-REM sleep compared to female subjects (72.0% ± 10.3% ETST vs. 75.5% ± 8.2% ETST, *p* = 0.08). Furthermore, in the age group >13 years the male subjects tended to greater VLFC compared to female subjects (31.2% ± 8.8% ETST vs. 27.8% ± 10.2% ETST, *p* = 0.08).

## Discussion

Assessing sleep parameters by means of the CPC technique revealed a clear increase of unstable sleep with progressing age as indicated by LFC and the estimated CAP-rate. At the same time also the amount disordered breathing (assessed by e-LFC), the amount of REM/wake (assessed by VLFC) states and the amount of sleep fragmentation increased. On the other hand, stable sleep as indicated by HFC and also the estimated proportion of non-REM sleep decreased with progressing age. Hence, all parameters consistently indicate a change in sleep quality that occurs most pronounced at the end of adolescence. This decrease is accompanied by a decrease of total sleep time with progressing age.

The average ETST showed a decline of approx. 150 min from infants to the end of adolescence. This decline is in accordance with the time course of total sleep time from polysomnographic recordings (Ohayon et al., 2004; McLaughlin Crabtree and Williams, 2009; Scholle et al., 2011; Carskadon and Dement, 2017). Hence, also the course of other sleep parameters (e.g., sleep period time, sleep efficiency, time spent in sleep stages N1–N3 and REM) should be comparable to previous studies even though we were not able to track them by the ECG based approach.

Generally, wake-up time should be constant throughout childhood and adolescence because the children had to be at school punctually. Hence, the decline in total sleep time with progressing age can only be explained by later bedtimes in the evening. This delay in bedtime is in accordance with the delay of the body clock by up to 2 h with progressing age (Roenneberg et al., 2004). In contrast, it has been shown that the sleep times are considerably longer on free days because the sleep amount is then determined by the body clock rather than the alarm clock, i.e., need to be at school in time (Roenneberg et al., 2012). The difference in sleep time caused by the difference of body or circadian clock and social clock is termed social jet lag and can be as large as 2 h (Wittmann et al., 2006; Roenneberg et al., 2012).

The estimated amount of non-REM sleep for the young children, i.e., pre-schoolers and primary schoolers is in accordance with the portion retrieved from polysomnographic recordings (approx. 80%) (Ohayon et al., 2004; Scholle et al., 2011). However, previous studies with polysomnographic recordings showed a proportion of non-REM sleep of approx. 75–80% also for adolescents (Ohayon et al., 2004; Scholle et al., 2011; Carskadon and Dement, 2017). Hence, the decrease of non-REM sleep time with progressing age estimated by HFC and LFC underestimates the amount of non-REM sleep for adolescents.

Unstable sleep is associated with the occurrence of CAP sequences and is quantified by LFC of CPC analysis. LFC increased during childhood and adolescence suggesting that the amount of CAP sequences during sleep increased during childhood and adolescence. LFC was below 10% for the pre-schoolers and increased to almost 20% at the end of adolescence. At the same time the estimated CAP-rate increased from 11 to 29% of ETST. The CAP-rate calculated from polysomnographic recordings was 25% for pre-schoolers and increased with progressing age (Parrino et al., 2012). At the age of approx. 15 years, the CAP-rate showed a peak (approx. 40%) and then slowly declined to approx. 35% at the age of 35. Neither the CAP-rate nor its local maximum were properly assessed by the estimated CAP-rate. The estimated CAP-rate generally underestimated the CAP-rate calculated from polysomnographic recordings. Furthermore, the more complex dynamics of the CAP-rate is not reflected by the estimated CAP-rate.

Very low frequency coupling as an indicator of REM/wake states increased with progressing age. REM and wakes states assessed from polysomnographic data show a constant [approx. 22% of sleep time (Ohayon et al., 2004)] or slightly decreasing amount of REM sleep [from approx. 25% to approx. 19% (Scholle et al., 2011)] during childhood and adolescence accompanied by a slight increase of wake after onset of sleep (from below 10 to 15%) (Ohayon et al., 2004). Hence, if these two numbers are added, VLFC seems to assess REM/wake states fairly well. The increase of VLFC with progressing age seems to be related to the increase of wake times after onset of sleep with progressing age.

Fragmented sleep or sleep disordered breathing is associated with the appearance of e-LFC (Thomas et al., 2007), which normally occurs around sleep onset, pre-REM sleep and at the junction of sleep cycles. Generally, the amount of respiratory disturbances during sleep increases from young adults to old age (Thomas et al., 2007; Pavlova et al., 2008). An increase of respiratory disturbances during childhood and adolescence has not been shown yet but seems to be plausible taking into account the present findings. The reliability of the approach of detecting sleep disordered breathing be mean of e-LFC is substantiated by the finding that a respiratory disturbance index based on LFC showed a good correlation with the respiratory disturbance index determined from polysomnographic recordings (Guo et al., 2011).

The amount of changes of sleep states between successive analysis windows, i.e., sleep fragmentation, increased with progressing age. A certain amount of changes of sleep state should be detectable because of the dynamics of sleep states. The present results suggest that the lower bound of fragmentation calculated by the present method should be approx. 20% on average.

As mentioned above, the social jetlag is responsible for the decline of the ETST. It should be investigated whether the decline of stable sleep and the increase of unstable sleep are associated with the delay of the body clock. I.e., sleep data acquired on free days should be investigated to control for effects caused by the social jet lag. Taken together, the data provide evidence that adolescent sleep is shorter, more unstable and, hence, poorer compared to sleep in early childhood. It remains to be clarified if this decline in sleep quality reflects normal maturational changes or if other factors such as, e.g., social jetlag contribute substantially to this finding. Furthermore, strategies should be developed that make adolescents aware of the positive effects of sleep to enable better self-management with respect to sleeping habits.

## Author Contributions

DC, GS, and FE conceived the study. GS and ML planned and managed the study. ML recruited subjects and was responsible for the ECG recordings. DC performed the data analysis. DC and FE drafted the manuscript. All authors edited and approved the manuscript.

## Conflict of Interest Statement

The authors declare that the research was conducted in the absence of any commercial or financial relationships that could be construed as a potential conflict of interest.
